# Enhancing gutta-percha with silver mesoporous calcium silicate nanoparticles for advanced endodontic applications

**DOI:** 10.1371/journal.pone.0329435

**Published:** 2025-08-12

**Authors:** Amer A. Mohammed, Ahmed H. Ali, Federico Foschi, Francesco Mannocci

**Affiliations:** 1 Aesthetic and Restorative Dentistry Department, College of Dentistry, University of Baghdad, Baghdad, Iraq; 2 Department of Restorative Dentistry, Unit of Endodontology, UCL Eastman Dental Institute, University College London, London, United Kingdom; 3 Department of Endodontics, Centre of Oral Clinical & Translational Sciences, Faculty of Dentistry, Oral & Craniofacial Sciences, Guy’s Dental Hospital, King’s College London, London, United Kingdom; Universidade Federal Fluminense, BRAZIL

## Abstract

**Aim:**

This study aimed to develop a modified gutta-percha (GP) multifunctional endodontic material by incorporating mesoporous calcium silicate nanoparticles (MCSNs) or silver-incorporated mesoporous calcium silicate nanoparticles (Ag-MCSNs) as bioactive nanoparticle fillers to enhance bioactivity, biomineralization, and radiopacity while achieving low cytotoxicity and improved antibacterial activity.

**Methodology:**

MCSNs and Ag-MCSNs were synthesized and incorporated into GP at concentrations of 1%, 5%, and 10% by weight. The chemical and structural characterization of the new GP materials was performed using Fourier-transform infrared spectroscopy (FTIR), X-ray diffraction (XRD), energy-dispersive spectrometry (EDS), and field emission-scanning electron microscopy (FE-SEM) to assess filler distribution. Biomineralization was evaluated using XRD, EDS, and scanning electron microscopy (SEM). Ion release, pH changes, MTT assays, and culture-based techniques were employed to assess bioactivity, biocompatibility, and antibacterial properties. Finally, radiopacity testing was conducted.

**Results:**

All samples of the newly developed GP exhibited uniform particle distribution within the GP matrix. Bioactivity and biomineralization tests revealed hydroxyapatite (HA) layer precipitation at 3, 7, 14, and 28 days, with maximum HA formation observed with prolonged immersion. Samples containing MCSNs or Ag-MCSNs created a weakly alkaline microenvironment initially, maintaining a suitable pH over time. All groups demonstrated non-cytotoxicity, with cell viability exceeding 70%. Antibacterial tests showed larger inhibition zones in all experimental groups compared to the control, with GP containing 10% MCSNs or Ag-MCSNs exhibiting the highest antibacterial activity, particularly Ag-MCSNs. Radiopacity tests indicated no significant difference between the experimental and control groups.

**Conclusion:**

Gutta-percha materials incorporated with 10% MCSNs or Ag-MCSNs demonstrated enhanced bioactivity, biomineralization, and antibacterial properties compared to the control GP, while maintaining suitable levels of biocompatibility in accordance with ISO 10993/2009 standards. The incorporation of Ag-MCSNs significantly improved antibacterial effects, particularly against *Enterococcus faecalis*, and increased radiopacity, making it a promising material for root canal therapy.

## Introduction

The primary objectives of endodontic therapy are to clean and shape the root canal system to allow effective disinfection and to fill the canal in three dimensions, creating a hermetic seal to prevent reinfection. [1,2]. Root canal treatments (RCTs) can be unsuccessful if the infected root canals are not properly sealed. This can lead to the regeneration of germs inside the root canal system, resulting in apical periodontitis and abscess formation. Despite the use of strong intracanal irrigants, such as 6% sodium hypochlorite (NaOCl), in root canal treatment (RCT) to eliminate microbial infection, achieving thorough disinfection of the intricate root canal anatomy is extremely challenging [3]. Despite advances in techniques, success rates for root canal treatment have stagnated over the past decades, with approximately 20% of cases failing to heal despite guideline-standard treatment as stated in studies. This has driven the development of new endodontic obturation materials aimed at improving long-term outcomes [4,5].

Gutta-percha (GP) has been widely used as a root canal obturation material in numerous clinical studies [6,7]. While GP offers advantages such as biocompatibility, making it less likely to provoke an immune response; cost-effectiveness; ease of removal facilitating retreatment; and a proven clinical track record, it is nonetheless limited by its inability to completely seal the root canal and prevent bacterial percolation, leading to reinfection and treatment failures [3,6,8]. For example, if the root canal seal is not sufficient and there are empty spaces within the filled root canal, there may be leakage because the gutta-percha material is not bonded to the dentinal surface and [9]. Void formation has been one of the most reported drawbacks considering the higher thickness of the sealer that needs to be applied [10]. Researchers have investigated various nanoparticles to address these challenges and explore their potential applications in drug delivery and antibacterial capabilities [4].

An ideal root canal filling material should possess antibacterial properties and promote mineral deposition in the apical area. In this context, mesoporous calcium silicate nanoparticles (MCSNs) are particularly well-suited due to their ability to promote apatite deposition, possess osteogenic properties, and demonstrate drug delivery and antibacterial capabilities [11]. Due to their highly organized mesoporous structures, offering controlled release kinetics and optimal surface area, these materials are effective carriers for antibiotics and antibacterial agents. Mesoporous materials have been studied extensively for their potential as drug delivery systems, offering controllable release kinetics [12]. Due to their highly organized mesoporous structures, offering controlled release kinetics and optimal surface area, these materials are effective carriers for antibiotics and antibacterial agents [13]. Additionally, MCSNs release Ca and Si ions, further enhancing their bioactivity [14]. In parallel, silver nanoparticles (AgNPs) are noted for their broad-spectrum antibacterial and antifungal properties; however, concerns about aggregation and cytotoxicity necessitate further evaluation. AgNPs exhibit effective antibacterial activity against *Enterococcus faecalis*, a common pathogen in endodontic infections. However, their tendency to aggregate and potential cytotoxicity remain concerns [15].

This study aimed to fabricate and characterize new GP-based root canal obturation materials enriched with MCSNs and Ag-MCSNs, which are tailored to enhance bioactivity, reduce cytotoxicity, and improve antibacterial properties.

## Materials and methods

### Fabrication of GP

GP points soft type 15 gm (Sure-endo, Sure Dent Corporation, Gyeonggi-do, Korea) were transferred into a glass beaker. The beaker was positioned inside an electric oven (Heat Carbolite, Hope Valley, UK) at 200°C for 15 minutes. Upon removal from the oven, the GP points were observed to become semi-soft. 5 ml of chloroform (Alpha Chemika, Mumbai, Maharashtra, India) was added into the beaker to dissolve the GP. The solvent was stirred continuously for 5 minutes at 25°C with a glass stick until the GP was completely dissolved into a liquid suspension [16,17].

### Fabrication of GP with MCSNs and Ag-MCSNs

MCSNs and Ag-MCSNs were synthesized and characterized in a previous study [18]. MCSNs and Ag-MCSNs (with three different weights representing three different filler percentages as 1%, 5%, and 10% sequentially for each type of filler) were dissolved in Tris-HCl (1M, pH 7.4) and stirred by sonication (Woodpecker DTE D2 LED Ultrasonic Scaler, China) for 30 min until all particles were dissolved [19]. The viscous material added to the previous dissolved GP was 1% (0.15 gm filler and 14.85 gm GP), 5% (0.75 gm filler and 14.25 gm GP), and 10% (1.5 gm filler and 13.5 gm GP), by using a magnetic stirrer each mixture was mixed until it reached a semi-viscous stage, after that the mixture was placed in glass Petri plates until completely dry and set 24 hours at room temperature (25°C).

### Fabrication of discs with customized device

A custom-designed device for pressurized material molding was used and fabricated to acquire materials discs that were 5 mm wide and 2 mm high. The design of the custom-made pressurized material molding device was added to the supplementary appendix (1).

### Molding of Gutta-percha discs

**Fig 1:**
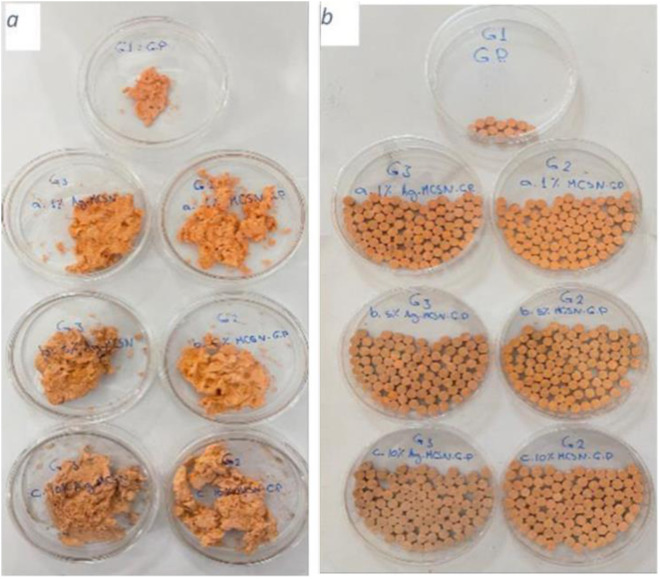
Groups of Gutta-percha. (a) Before molding, and (b) after molding.

0.15gms of materials was weighed by 3 digits’ digital jewelry scale (YD-9099, China), and added to the Pressing room of the fabricated device, and a constant pressure of 150 bar was applied for 10 seconds to the materials to create a homogeneous disk for all groups, as shown in (Fig 1).

### Material characterization using FTIR, XRD, EDS, and FESEM

This study was performed by using an Fourier-transform infrared spectroscopy FTIR device (FTIR, Shimadzu, Japan.) to analyze the chemical composition of the tested materials, confirming the incorporation of MCSNs and Ag-MCSNs fillers into gutta-percha (GP). GP samples were ground into a fine powder and mixed with potassium bromide (KBr) powder at a 1:100 ratio until a homogeneous mixture was achieved [20]. Compressed thin pellets were formed using a pellet press for FTIR measurement in an absorption mode (the scan range 400−4000 cm^-1^). (Shimadzu, Japan) under a pressure of 13.8–17.2 MPa [21].

The samples were analyzed using X-ray diffraction (XRD) over a range of 5–80 degrees 2θ with Cu-Kα radiation. Initial testing was conducted on control materials, followed by analysis of the newly developed GP materials containing MCSNs fillers (1%, 5%, and 10%) and Ag-MCSNs fillers (1%, 5%, and 10%). For each specimen, the diffraction patterns were recorded continuously at a scanning speed of approximately 5°/min. The XRD analysis was performed using a Shimadzu LabX XRD-6000 system (Japan).

Energy-dispersive X-ray spectroscopy (EDS) was employed for elemental analysis and chemical characterization of both the control and experimental GP materials containing MCSNs fillers (1%, 5%, and 10%) and Ag-MCSNs fillers (1%, 5%, and 10%). The analysis was conducted using an EDS-equipped SEM system (Axia ChemiSEM, USA).

Field emission scanning electron microscopy (FESEM) observations were carried out to examine the homogeneity of the experimental GP materials and the distribution of fillers within the GP matrix. Samples measuring 5 mm in width and 2 mm in thickness were prepared for FESEM analysis. The FESEM study was conducted using a CSEMFEG Inspect F50 system (USA) under varying magnifications, including 2 kx, 4 kx, 7 kx, 13 kx, 25 kx, 50 kx, and 100 kx.

### Biomineralization test of new GP

The biomineralization activity was evaluated in vitro using control and experimental GP discs containing MCSNs fillers (1%, 5%, and 10%) and Ag-MCSNs fillers (1%, 5%, and 10%), developed during this study. Simulated body fluid (SBF), mimicking the ion concentration of human blood plasma, was freshly prepared with the following composition: Na^+^ 142.0, K^+^ 5.0, Ca^2+^ 2.5, Mg^2+^ 1.5, Cl^-^ 147.8, HCO_3_^–^4.2, HPO_4_^2–^1.0, and SO_4_^2^–0.5 mM The pH was adjusted to 7.4 using hydrochloric acid [17]. The samples were soaked in 100 mL of SBF at 37°C in an incubator (Faithful Instrument (Hebei), China) for 3, 7, 14, and 28 days [11,19,22–24]. At each time point, three discs from each group were removed for biomineralization analysis. The samples were rinsed with distilled water to eliminate any soluble ions.

The structure and composition of the films formed on the surfaces were analyzed using XRD, EDS, and SEM. Crystalline phases of the samples were examined using an XRD system (Shimadzu LabX XRD-6000, Japan) with Cu-Kα radiation over a 5–80° 2θ range. The diffraction patterns for each specimen were recorded continuously at a scanning speed of approximately 5°/min. The mineral crystal formation on the sample surfaces was assessed using energy-dispersive X-ray spectroscopy (EDS, SEM-Axia ChemiSEM, USA). A Scanning Electron Microscope (SEM-Axia ChemiSEM, USA) was employed to evaluate the formation of mineral crystals on the sample surfaces at magnifications of 0.8 kx, 1.5 kx, 3 kx, 6 kx, 12 kx, and 25 kx.

### Ion release and pH evaluation of new GP

The samples were immersed in 100 mL of SBF at 37°C in an incubator for 3, 7, 14, and 28 days. SBF solutions were collected to assess variations in ion concentrations. Group 1 (control) was analyzed for P ions, group 2 (GP with MCSNs) was analyzed for Ca, Si, and P ions, and group 3 (GP with Ag-MCSNs) was analyzed for Ca, Si, Ag^+^, and P ions. At each time point, 5 mL of the solution was extracted three times per sample. Ion concentrations were determined using inductively coupled plasma optical emission spectrometry (ICP-OES, PlasmaQuant 9100 series, Analytik Jena, Germany). pH changes over time were measured at 3, 7, 14, and 28 days using a pH meter (HI 2550 pH/ORP & EC/TDS/NaCL Meter, HANNA Instruments, USA).

### Cytocompatibility study of the new GP

The cytotoxicity were tested indirectly by Methyl Thiazol Tetrazolium (MTT) assay (indirect contact test by exposing the cells to the nanoparticles using the Human Dermal Fibroblast cells as cell lines). Primary Human Dermal Fibroblasts (HDFs) were obtained commercially from ATCC, and cultured according to the supplier’s protocol. Primary human dermal fibroblast cells were cultured in RPMI 1640 medium supplemented with 10% fetal bovine serum and 1% penicillin/streptomycin. Cells from passages 7–14 were used. The MTT assay was performed in accordance with ISO 10993-5:2009 standards to evaluate cytotoxicity of materials. Cells (1 × 10⁴) were seeded in 96-well plates and incubated with test materials. After incubation, MTT solution was added, followed by solubilization of formazan crystals. Absorbance was measured at 570 nm, and cell viability was calculated as a percentage relative to control wells [25]. The test was conducted at 24, 72, and 168 hours for each interval, with three replicates per time point. Both control and experimental GP discs containing MCSNs fillers (1%, 5%, and 10%) and Ag-MCSNs fillers (1%, 5%, and 10%) were evaluated.

### Antimicrobial study of new GP

This microbiological study was conducted to evaluate the antibacterial activity (inhibition zone) against *Enterococcus faecalis* of the experimental material (New GP) using discs measuring 2 mm in height and 5 mm in width. The *Enterococcus faecalis* strain was maintained in frozen stock cultures in brain heart infusion (BHI) broth. The colonies of *E. faecalis* were cultured in BHI broth at 37°C for 24 hours [26]. The bacterial strain used in this study was *Enterococcus faecalis* (ATCC 29212; American Type Culture Collection, Manassas, Virginia, USA).

Six Petri dishes were prepared using the agar well diffusion assay, with one serving as the control and the others for each experimental material [27]. The plates were incubated aerobically at 37°C for 24 hours. The following day, the agar plates were examined for bacterial inhibition zones. The diameters of these zones were measured using a digital Vernier calliper (Mitutoyo America Corporation, USA), ensuring precision by aligning the Vernier calliper through the center of the wells.

### Evaluation of radiopacity

The radiopacity of the tested materials was evaluated using dental radiography equipment (Genoray PORT-X4, Korea) combined with a digital phosphor plate system (Kavo, Germany) and a grayscale aluminium step wedge made from 6061 aluminium alloy (97.9% pure). The step wedge, with consistent density throughout the raw material, consisted of 10 steps, each 10 mm wide. The height of the first step was 1 mm, with subsequent steps increasing by 1 mm in height, creating a gradient from 1 mm to 10 mm.

The grayscale values ranged from 0 (black) to 255 (white), and the measurements were converted into millimetre equivalents of aluminium. Radiographic parameters included 70 kV, 8 mA, and an exposure time of 0.20 seconds. The object-to-focus distance was fixed at 22 cm (ANSI/ADA 2000) using a mold. Radiopacity was determined by comparing the grayscale measurements of the tested GP samples with ten replicates with the aluminium step wedge, which had thicknesses ranging from 1 mm to 10 mm in 1 mm increments (ANSI/ADA 2000 Specification No. 57) [28]. Radiographic images were analyzed on a computer monitor using ImageJ software (ImageJ Processing and Analysis 1.54d, Java Version 1.80) [29,30].

**Inclusivity in global research:** Additional information regarding the ethical, cultural, and scientific considerations specific to inclusivity in global research is included in the Supporting Information (S1 Checklist)

### Statistical analysis

The statistical analysis involved assessing the normal distribution of data using the Shapiro-Wilk test. Descriptive statistics are provided as means with corresponding standard deviations (SD) for each experimental group. All statistical analyses were conducted using SPSS software, version 26.0 (SPSS Inc., Chicago, IL, USA), ensuring rigorous computational analysis. A one-way ANOVA was performed to assess statistical differences across the multiple experimental groups, based on the assumption of homogeneity of variances. To determine specific intergroup differences following a significant ANOVA result, both Tukey’s and Dunnett’s post hoc tests were utilized, with the former examining all pairwise comparisons and the latter specifically comparing each treatment group to a control. A significance level of p < 0.05 was considered statistically significant.

## Results

### Chemical analysis of the new Gutta-Percha

The FTIR spectra for GP show characteristic peaks for O-H, CH2, C = O, CO3^2^⁻, C-O, C-H, and Zn-O bonds. The FTIR analysis of GP with MCSNs revealed energy changes across different bands. The Si–O–Si band, absent in the control GP, appeared at 875.68 cm^−1^ for 1% filler and at 867.97 cm^−1^ for 5% filler, while for 10% MCSNs fillers, the band shifted further. The Si-OH band was absent in the control group but appeared at 3749.62 cm^−1^ for the 1% filler and at 3751.55 cm^−1^ for both the 5% and 10% MCSNs fillers.

**Fig 2:**
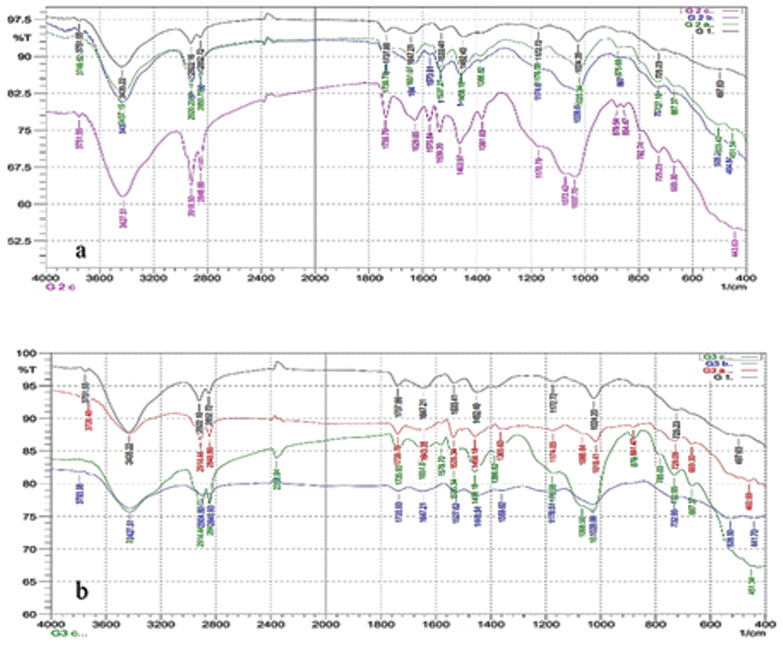
FTIR for (a) Gutta-percha and Gutta-percha with 1%,5% and 10% Ag-MCSNs and (b) for Gutta-percha and Gutta-percha with 1%, 5% and 10%MCSNs.

The FTIR spectra for GP with Ag-MCSNs revealed similar energy shifts. The Ag–O band, absent in the control group, appeared at 460.99 cm^−1^ and 1365.6 cm^−1^ for 1% filler, at 441.7 cm^−1^ and 1359.82 cm^−1^ for 5% filler, and at 451.34 cm^−1^ and 1386.82 cm^−1^ for 10% fillers. The Si–O–Si band appeared at 881.47 cm^−1^ for both the 1% and 5% fillers and at 879.54 cm^−1^ for the 10% filler. Similarly, the Si-OH band appeared at 3728.4 cm^−1^ for the 1% filler and at 3793.98 cm⁻¹ for both the 5% and 10% fillers (Fig 2).

The crystalline nature of GP was confirmed through X-ray diffraction analysis. The XRD pattern of GP revealed a crystalline structure with an amorphous hump attributable to MCSNs (1%, 5%, and 10%) and Ag-MCSNs (1%, 5%, and 10%). The analysis identified zincite as the major component of GP, along with other oxides like magnesium, zirconium, titanium, calcium silicate, and silver.

EDS analysis revealed the presence of C, O, Mg, Ti, Ni, Zn, and Zr in control GP. In GP with MCSNs, additional elements such as Si, Cl, and Ca were detected, while GP with Ag-MCSNs showed the presence of Ag in addition to these elements. Table 1 shows the ratios and percentages of these elements in each material.

**Table 1:** Elements proportion of Gutta Percha with 10% Ag- MCSNs according to EDS analyses.

Element	Atomic %	Atomic % Error	Weight %	Weight % Error
C	54.5	3.6	27.9	1.8
O	21.8	2.2	14.9	1.5
Si	6.6	0.4	7.8	0.5
Ca	0.7	0.1	1.2	0.2
Zn	14.1	0.8	39.1	2.3
Zr	2.1	1.1	8.0	4.1
Ag	0.2	0.1	1.0	0.5

**Fig 3:**
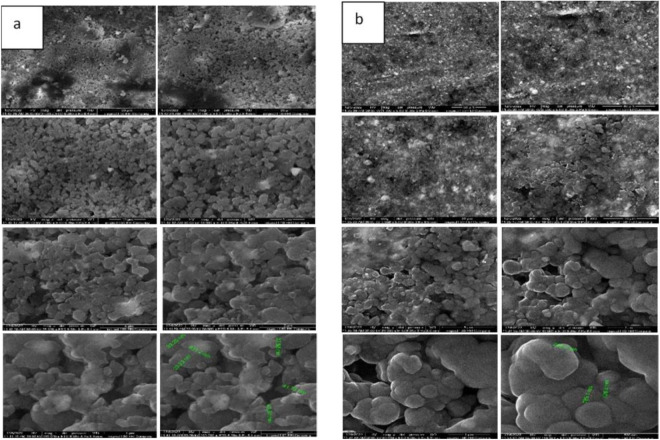
FE- SEM for (a) Gutta-percha with 10% MCSNs and (b) Gutta-percha with 10% Ag-MCSNs under different magnifications (2 kx,4 kx, 7 kx, 13 kx, 25 kx, 50 kx, and 100 kx).

Field emission-scanning electron microscopy (FE-SEM) provided high-resolution images of the GP surfaces, clearly showing the uniform distribution of MCSNs and Ag-MCSNs fillers across all concentrations. The fillers’ diameters ranged from 206.7 nm to 732.0 nm for MCSNs and from 25.07 nm to 55.7 nm for Ag-MCSNs. As the filler concentration increased, agglomeration was observed, resulting in a reduction in the GP matrix, potentially impacting its properties, as shown in (Fig 3)

### Biomineralization tests of new GP

**Fig 4:**
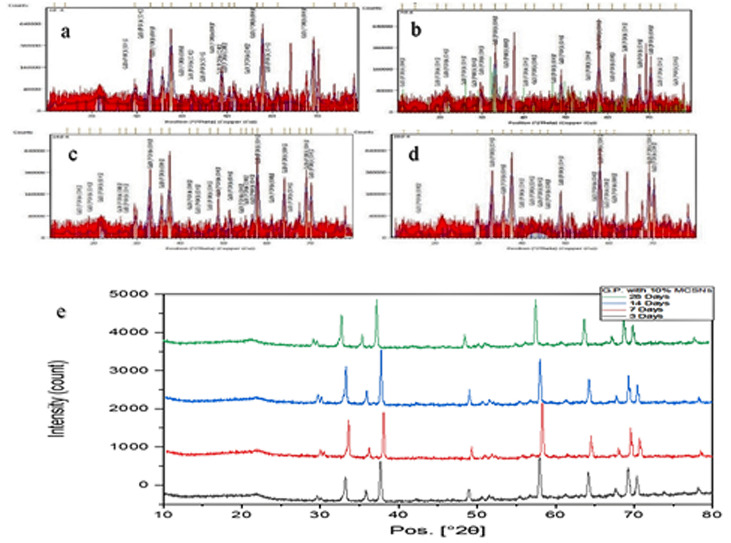
X.ray diffraction (XRD) for Gutta-percha after mixing with 10% MCSNs filler. (a) after 3 days, (b) after 7 days, (c) after 14 days, (d) after 28 days using X’pert highscore software – 23 & (e) XRD different days.

**Fig 5:**
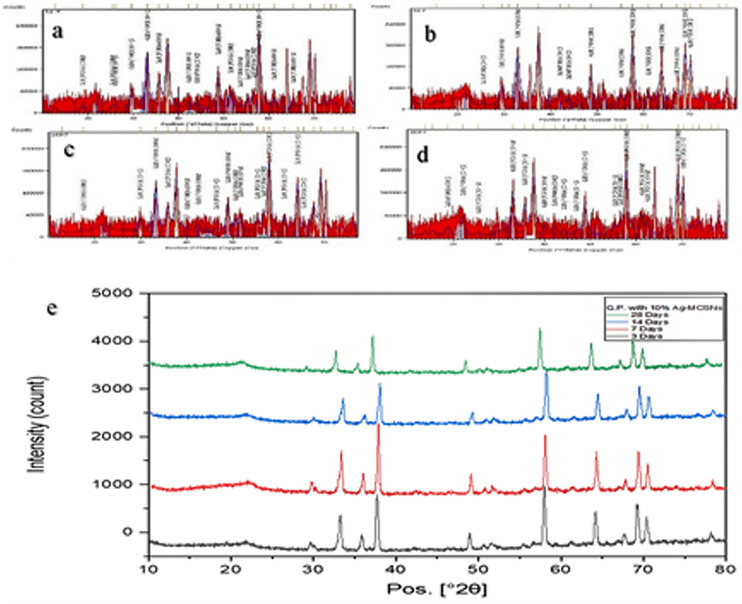
X.ray diffraction (XRD) for Gutta-percha after mixing with 10% Ag-MCSNs filler. (a) after 3 days, (b) after 7 days, (c) after 14 days, (d) after 28 days using X’pert high score software – 23 & (e) XRD different days.

XRD analysis of control GP samples before mixing revealed no evidence of hydroxyapatite (HA) formation. However, GP samples with MCSNs and Ag-MCSNs fillers displayed characteristic HA peaks, indicating HA formation on the surface. With prolonged soaking, HA peak intensities increased, demonstrating a rise in HA content specially for 10% filler samples as shown in Figs 4 and 5.

**Fig 6:**
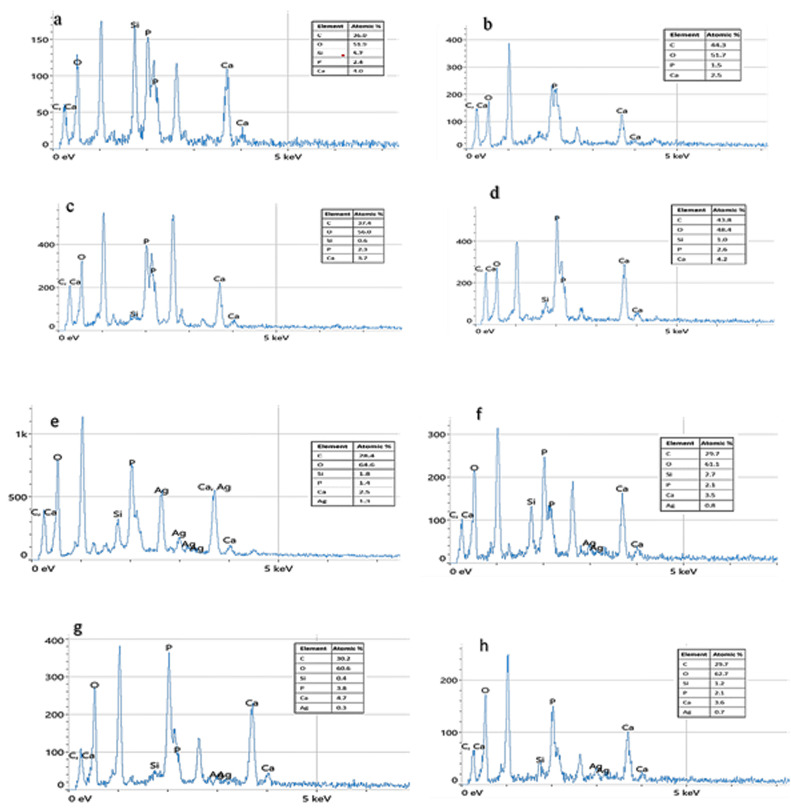
EDS spectrum of Gutta-percha with 10% MCSNs for the apatite layer formation after soaking in SBF for (a). 3 days, (b). for 7 days, (c). for 14 days, (d). for 28 days, 10% Ag-MCSNs for the apatite layer formation after soaking in SBF for (e). 3 days, (f). for 7 days, (g). for 14 days & (h). for 28 days.

EDS analysis confirmed minimal calcium (Ca) and phosphorus (P) ions in the control GP, with Ca/P ratios ranging between 0.05–0.4. However, GP with MCSNs fillers showed Ca/P ratios of 1.60–1.71, and GP with Ag-MCSNs fillers showed ratios of 1.55–1.78, approximating the theoretical stoichiometry of HA (Ca/P = 1.67) as in (Fig 6)

**Fig 7:**
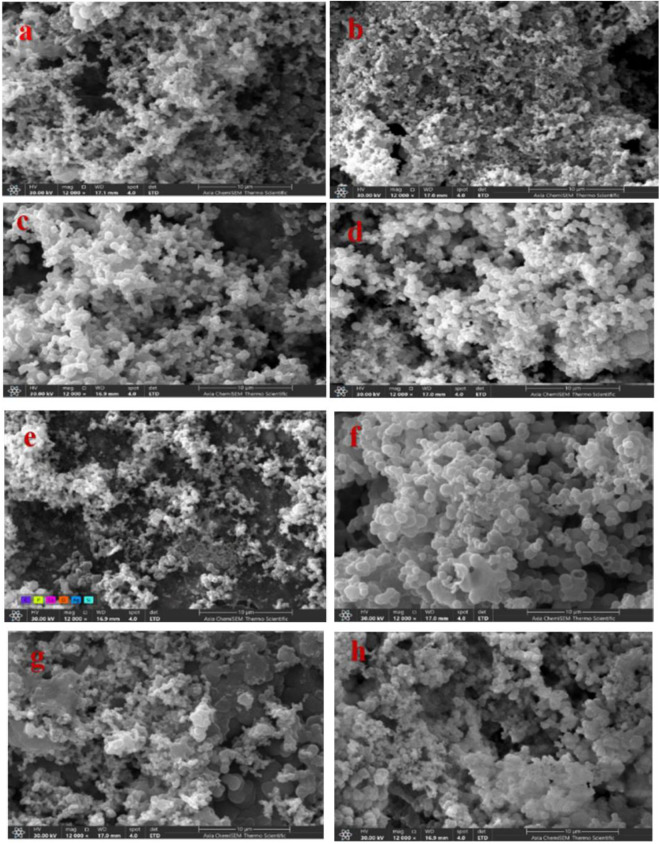
Scanning electron microscope (SEM) for Gutta-percha with 10% MCSNs after soaking in SBF for (a) 3 days, (b)7 days, (c)14 days, (d) 28 days, Gutta-percha with 10% Ag-MCSNs after soaking in SBF for (e)3 days, (f)7 days, (g) 14 days & (h) 28 days with 12 kx magnification.

SEM micrographs indicated that pure GP lacked significant apatite formation over 3, 7, 14, and 28 days, demonstrating poor bioactivity compared to GP with nano-fillers. The surfaces of GP samples with MCSNs and Ag-MCSNs fillers exhibited spherical HA nanoparticles after immersion in SBF. Over time and with higher filler concentrations, more HA particles formed, leading to a denser and more compact surface structure specially for 10% filler samples as shown in (Fig 7)

### Ion release and pH evaluation of new GP

**Fig 8:**
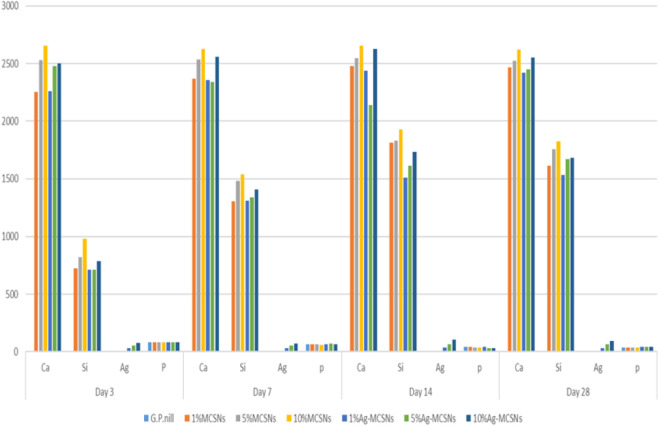
Bar chart of ion release profile of Gutta-percha, MCSNs, and Ag-MCSNs with 1%, 5% &10% filler.

**Fig 9:**
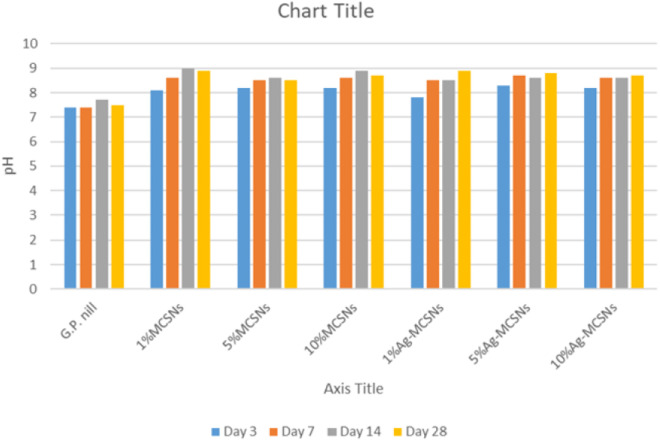
Bar chart of pH measurement of Gutta-percha, MCSNs, and Ag-MCSNs with 1%, 5% &10% filler.

Figs. 8 and 9 illustrate the Ca, Si, Ag, and P ion concentrations in SBF solutions, measured by ICP-OES after immersion of gutta-percha (GP) samples with various filler concentrations. The release rates of Ca, Si, and Ag increased progressively with higher concentrations of MCSNs and Ag-MCSNs. GP containing 10% MCSNs released Ca ions at 58.2 ± 4.1 ppm, significantly more than the 1% (33.5 ± 3.7 ppm) and 5% (46.9 ± 4.5 ppm) groups (p < 0.05). Similarly, 10% Ag-MCSNs exhibited the highest Ag ion release (26.4 ± 2.8 ppm), which was significantly greater than the 1% (10.7 ± 1.9 ppm) and 5% (18.3 ± 2.2 ppm) Ag-MCSNs groups (p < 0.01). This increase was observed specifically in the 10% Ag-MCSNs group, showing markedly higher elemental ion release than the control (p < 0.01).

**Fig 10:**
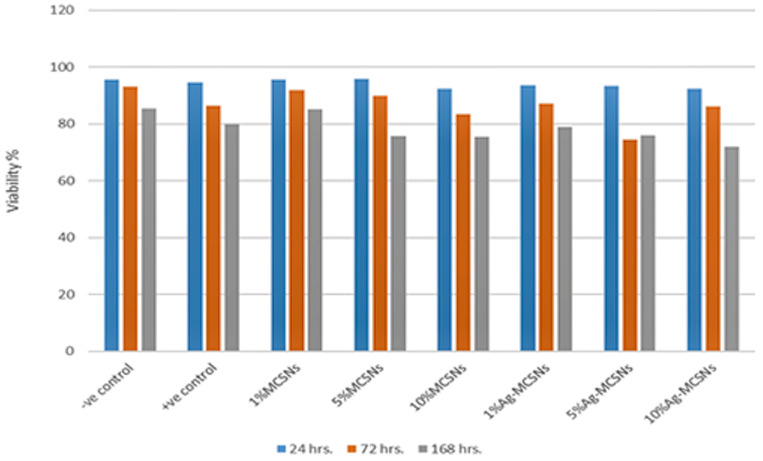
Bar chart of MTT assay test on the HDFn Cell of all experimental groups at 24, 72, and 168 hrs.

Throughout the soaking period, all samples maintained an alkaline pH near 8.5. GP with 10% MCSNs showed a slightly higher stabilized pH of 8.6 ± 0.2, compared to 8.3 ± 0.1 in the 10% Ag-MCSNs group, indicating a stronger buffering effect that supports apatite formation (Fig. 10). Initially, all groups induced a weakly alkaline environment, which stabilized by day 7.

### Cytocompatibility study of new GP

The MTT assay results demonstrated that GP samples containing MCSNs or Ag-MCSNs fillers (1%, 5%, and 10%) showed no cytotoxic effect on HDFn cells at 24, 48, and 72 hours. All groups maintained cell viability above 70%, with values ranging from 74.3% ± 2.5% to 92.1% ± 2.1%, confirming non-cytotoxicity based on ISO 10993-5 criteria (Fig. 10). GP with 10% MCSNs exhibited the highest cell viability at 72 hours (92.1% ± 2.1%), significantly higher than the 10% Ag-MCSNs group (81.4% ± 1.8%, p < 0.01). Similarly, at 48 hours, GP with 5% MCSNs (89.7% ± 1.9%) showed significantly greater viability than 5% Ag-MCSNs (77.3% ± 2.4%, p < 0.01). Notably, no significant difference was observed between the 1% MCSNs group (84.5% ± 2.3%) and the control (83.8% ± 2.6%) at 168 hours (p > 0.05), supporting the biocompatibility of lower filler concentrations over extended exposure. This increase in metabolic activity was observed specifically in the 1% MCSNs group, which showed the highest viability among all tested groups at all-time points (p < 0.01 compared to 10% Ag-MCSNs and control).

### Antimicrobial activity of the new GP

**Fig 11:**
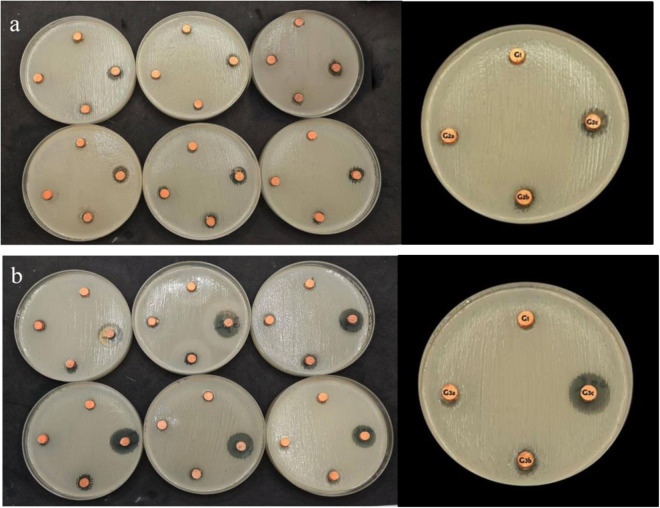
Inhibition zones against Enterococcus faecalis of (a) control group, Gutta-percha with 1%, 5% & 10% MCSNs and (b) control group, Gutta-percha with 1%, 5% & 10% Ag-MCSNs.

**Fig 12:**
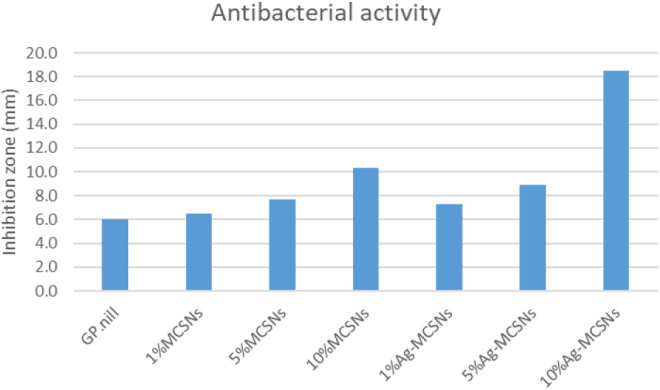
Bar chart showing the mean values of inhibition zones of all groups against Enterococcus faecalis.

The antimicrobial study revealed that GP samples containing Ag-MCSNs fillers exhibited significantly larger inhibition zones compared to those with MCSNs fillers. Specifically, the 10% Ag-MCSNs group showed the highest antibacterial activity, with an inhibition zone of 15.2 ± 0.6 mm, which was significantly larger than the 5% Ag-MCSNs group (12.8 ± 0.5 mm), the 1% Ag-MCSNs group (9.4 ± 0.7 mm), and all MCSNs-based groups (p < 0.01). This increase was observed specifically in the 10% Ag-MCSNs group, larger than the control (6.3 ± 0.4 mm, p < 0.01). Among the MCSNs groups, only the 5% and 10% concentrations showed moderate antibacterial activity (inhibition zones of 8.7 ± 0.5 mm and 10.1 ± 0.6 mm, respectively), while the 1% MCSNs group (6.5 ± 0.3 mm) did not differ significantly from the control. These findings are visually represented in Figs 11 and 12.

### Evaluation of radiopacity

**Fig 13:**
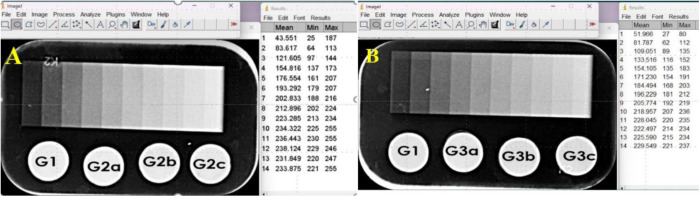
Radiopacity evaluation (grayscale) (a) for the control group, G.P. with 1%, 5%, and 10% MCSNs and (b) for control group, G.P. with 1%, 5%, and 10% Ag-MCSNs (b).

The Radiopacity analysis revealed no significant difference in grayscale values between the control group and experimental groups. Minimum, maximum, mean, and standard deviation values of grayscale measurements confirmed comparable radiopacity levels across all samples, as shown in Table 2 and Fig 13.

**Table 2:** Means, SD, SE, 95% CI, minimum, and maximum of grayscale measurements of new gutta-percha.

	N	Mean	Std. Deviation	Std. Error	Minimum	Maximum	p-value^*^
1% MCSNs	10	9.9650	.67360	.21301	8.09	10.42	0.318
5% MCSNs	10	9.8820	.78864	.24939	8.09	11.40	
10%MCSNs	10	9.9710	1.18163	.37366	7.88	12.80	
1%Ag-MCSNs	10	10.3110	.12467	.03943	10.16	10.50	
5%Ag-MCSNs	10	10.3030	.08908	.02817	10.13	10.44	
10%Ag-MCSNs	10	10.4040	.10885	.03442	10.21	10.56	
Control	10	10.2505	.19652	.06214	9.77	10.45	
Total	70	10.1552	.60829	.07271	7.88	12.80	

* ANOVA.

## Discussion

Acknowledge the potential for similar studies, this study is among the first to investigate the incorporation of MCSNs combined with Ag-MCSNs into gutta-percha (GP) as an active biomaterial. To the best of our knowledge, this is the first study to incorporate mesoporous calcium silicate nanoparticles (MCSNs) combined with silver nanoparticles (Ag-MCSNs) into gutta-percha (GP) as an active biomaterial. This innovative material aims to develop an obturating material with enhanced antibacterial, bioactive, and biocompatible properties.

### Chemical analysis

According to the FTIR analysis conducted in this study, the incorporation of MCSNs and Ag-MCSNs did not induce any detectable chemical changes in the gutta-percha (GP) matrix, as the characteristic peaks corresponding to both the nanoparticles and the original GP remained in similar positions without significant shifts. These results are in agreement with previous reports indicating the stability of GP’s chemical structure upon nanoparticle incorporation [31]. Furthermore, our XRD findings confirmed the homogeneity of the modified material, as no new diffraction peaks appeared from the added components, suggesting successful integration. The formation of broad, low-intensity patterns corresponding to amorphous phases, observed particularly in the presence of MCSNs and Ag-MCSNs, further indicated a uniform distribution of the fillers within the GP matrix—an outcome that is consistent with earlier studies on similar nanocomposites [32].

EDS analysis provided detailed information on the elemental composition of the GP samples. The addition of MCSNs increased the content of Ca and Si, while Ag-MCSNs samples showed higher Ca, Si, and Ag levels. The observed increase in Ca, Si, and Ag content was due to the intentional higher nanoparticle inclusion, not merely analysis parameters like x-ray power or temperature used during analysis [33]. FE-SEM imaging confirmed the uniform distribution of fillers in GP, with larger filler concentrations (5% and 10%) displaying better incorporation compared to 1% fillers. The high particle distribution observed in this study is a key advantage of the newly developed material.

### Biomineralization properties

XRD analysis did not detect hydroxyapatite (HA) formation in the control group under the conditions tested, whereas GP samples with MCSNs and Ag-MCSNs exhibited HA peaks, confirming HA formation on their surfaces. The HA content increased with higher filler percentages and prolonged incubation periods [34].

EDS analysis revealed that GP samples with both MCSNs and Ag-MCSNs demonstrated superior apatite mineralization capabilities compared to the control group, which lacked sufficient Ca^2^⁺ and SiO_4_^4−^ ions for biomineralization. SEM imaging supported these findings, showing crystal deposition layers on the surface of GP samples with 10% fillers, while the control group exhibited poor bioactivity. The dense morphology of HA formed with increasing filler content highlights its potential for improving apical seals and preventing bacterial penetration [35].

ICP analysis demonstrated that the release rates of Ca, Si, and Ag ions increased with higher concentrations of MCSNs and Ag-MCSNs fillers. GP with MCSNs achieved steady ion release within seven days, while GP with Ag-MCSNs required up to 14 days to stabilize. The recorded decrease in P ion concentration in SBF is attributed to their incorporation into the hydroxyapatite structure formed on GP surfaces with fillers [36].

The pH of the solutions remained alkaline (~8.5), with GP samples containing MCSNs exhibiting slightly higher pH levels compared to Ag-MCSNs. The reduced pH of Ag-MCSNs samples was attributed to Ag⁺ binding to Si⁴ ⁺ skeletons, altering the release rates of Si⁴ ⁺ ions [23]. Notably, the denser HA morphology observed with higher filler content could contribute to more effective prevention of bacterial infiltration at the critical apical region.

### Cytocompatibility study

The MTT assay results showed higher cell viability for GP with MCSNs compared to GP with Ag-MCSNs. The rapid release of Ag⁺ ions was associated with increased cytotoxicity, which was time-dependent and concentration-dependent [37]. Although no significant differences were observed in cell viability within the first 24 hours, notable differences emerged by 48 and 72 hours. High pH levels, resulting from Ca(OH)₂ formation, potentially inhibited cell proliferation, aligning with findings from prior studies [38,39]. The sustained release of Ca^2^⁺ and SiO₃^2^⁻ ions contributed to tissue regeneration and enhanced biocompatibility [40,41]. According to ISO 10993/2009 standards, all modified GP samples were classified as non-cytotoxic, with cell viability exceeding 70%. Clinically, these findings indicate that the modified gutta-percha could promote favorable biological responses necessary for long-term tissue integration, provided that ion release profiles are optimized.

### Antimicrobial study

The main goal of chemo mechanical endodontic treatment is the reduction or elimination of microorganisms from the root canal system [42]. GP with Ag-MCSNs demonstrated superior antibacterial efficacy compared to GP with MCSNs. The antibacterial properties of Ag-MCSNs were attributed to Ag⁺ ions, which damage bacterial cell walls and membranes, leading to cell lysis and intracellular disruptions [43–45]. The antibacterial efficacy of Ag-MCSNs likely results from interactions with bacterial membranes and subsequent intracellular disturbances [18,46]. Modified material capable of exerting sustained antimicrobial effects at the site of obturation could provide an additional defense against persistent infection, enhancing the prognosis of root canal therapy.

### Evaluation of radiopacity

Radiopacity testing revealed no significant differences in grayscale values between control and experimental groups. The inclusion of MCSNs and Ag-MCSNs fillers achieve comparable radiopacity aligning with international standards for root canal filling materials [47,48].

## Conclusion

This study demonstrates that the newly developed GP incorporating MCSNs and Ag-MCSNs, particularly at a 10% concentration, exhibits superior bioactivity, biomineralization, and antibacterial properties compared to control GP. The addition of MCSNs and Ag-MCSNs did not negatively impact biocompatibility, with all samples classified as non-cytotoxic. GP with Ag-MCSNs demonstrated the highest antibacterial efficacy against Enterococcus faecalis and comparable radiopacity. These findings underscore the potential of GP with Ag-MCSNs as an advanced root canal filling material, potentially improving treatment outcomes by enhancing antimicrobial action and biomineralization.

### Limitations and recommendations

While the initial findings are promising, the study has limitations. The antibacterial assessment focused only on Enterococcus faecalis, which is a primary endodontic pathogen but does not represent the full spectrum of microbial species involved in endodontic infections. The findings are limited to laboratory experiments and require validation through clinical trials to confirm their effectiveness and safety in real-world endodontic applications. Further studies, including clinical evaluations and extended biocompatibility assessments, are necessary to confirm the potential of the newly developed gutta-percha material for routine clinical use.

## Supporting information

S1 Appendix and Fig This ai the S1 appedix (Design the test pressure system (TPS)) and Fig. Designing of the custom-made pressurized material molding device.
(DOCX)

S1 Dataset This is the S1 dataset available for antibacterial activity, cytotoxicity and radiopacity. (DOCX)

S1 Protocol This is the S1 protocol of MTT assay procedure. (DOCX)

S1 Fig This is the S1 Fig of SEM for G.P. (DOCX)

S2 Fig This is the S2 Fig of XRD spectra for G.P. mixed with MCSNs and Ag-MCSNs. (DOCX)

S1 Checklist Inclusivity in global research. (DOCX)
