# Supplementary material for: Enhancing gutta-percha with silver mesoporous calcium silicate nanoparticles for advanced endodontic applications
Source: PLoS One. 2025 Aug 12;20(8):e0329435. doi: 10.1371/journal.pone.0329435 (PMC12342242; doi:10.1371/journal.pone.0329435)
Supplement: S1 Appendix and Fig — Designing of the custom-made pressurized material molding device. (DOCX) [file pone.0329435.s001.docx]

**S1 appendix (Design the test pressure system (TPS)) and Fig. Designing of the custom-made pressurized material molding device.**

The system was designed and developed using the SolidWorks program, which is internationally recognized for professional design. The system was designed according to the specific metal type suitable for the purpose. Steel is the recommended base metal for designing and manufacturing the structure due to its ability to withstand high pressure. Additionally, the remaining parts of the system were designed to follow international standards. It was important to use a drawing scale of 1:1 to achieve accurate measurements of the pressing chamber. The innovative system design has been proven effective in practical applications and has become an essential testing platform for conducting multiple tests. The design of the custom-made pressurized material molding device is shown in Fig 1


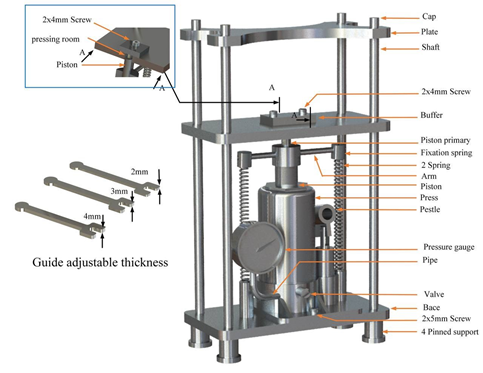

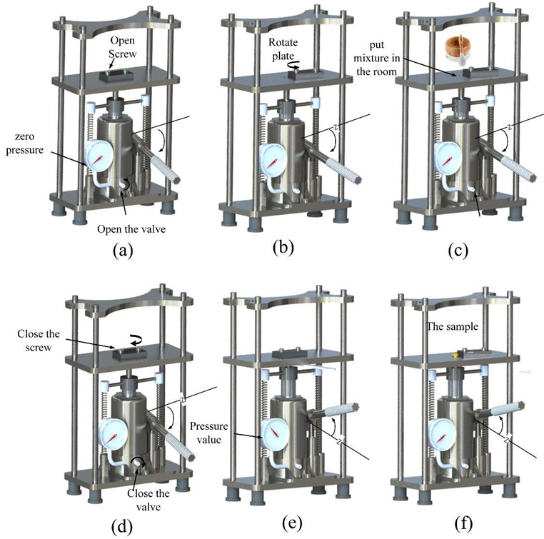


**Figure: Designing of the custom-made pressurized material molding device.**
