# Supplementary material for: Enhancing gutta-percha with silver mesoporous calcium silicate nanoparticles for advanced endodontic applications
Source: PLoS One. 2025 Aug 12;20(8):e0329435. doi: 10.1371/journal.pone.0329435 (PMC12342242; doi:10.1371/journal.pone.0329435)
Supplement: S1 Dataset — (DOCX) [file pone.0329435.s002.docx]

**S2 Dataset**

1. **Antibacterial activity**

| Inhibition zone (mm) | | | | |
| --- | --- | --- | --- | --- |
| Group | **Mean** | **Std. Deviation** | **Minimum** | **Maximum** |
| GP. | 6.1 | 0.1 | 6.0 | 6.2 |
| 1%MCSNs | 6.5 | 0.2 | 6.2 | 6.8 |
| 5%MCSNs | 7.7 | 0.3 | 7.3 | 8.0 |
| 10%MCSNs | 10.3 | 0.6 | 9.3 | 11.0 |
| 1%Ag-MCSNs | 7.3 | 0.3 | 7.0 | 7.6 |
| 5%Ag-MCSNs | 8.9 | 0.2 | 8.5 | 9.2 |
| 10%Ag-MCSNs | 18.5 | 0.3 | 18.2 | 19.0 |

| ANOVA | | | | | |
| --- | --- | --- | --- | --- | --- |
| Inhibition zone (mm) | | | | | |
|  | Sum of Squares | df | Mean Square | F | Sig. |
| Between Groups | 662.942 | 6 | 110.490 | 1091.392 | .000 |
| Within Groups | 3.543 | 35 | .101 |  |  |
| Total | 666.486 | 41 |  |  |  |

| Dependent Variable: | | | | | | |
| --- | --- | --- | --- | --- | --- | --- |
| Tukey HSD | | | | | | |
|  |  |  |  |  | **95% Confidence Interval** | |
| (I) Group | **(J) Group** | **Mean Difference (I-J)** | **Std. Error** | **p-value** | **Lower Bound** | **Upper Bound** |
| GP.nill | 1%MCSNs | -0.383 | 0.184 | 0.382 | -0.958 | 0.191 |
|  | 5%MCSNs | -1.56667^*^ | 0.184 | 0.000 | -2.141 | -0.992 |
|  | 10%MCSNs | -4.20000^*^ | 0.184 | 0.000 | -4.774 | -3.626 |
|  | 1%Ag-MCSNs | -1.23333^*^ | 0.184 | 0.000 | -1.808 | -0.659 |
|  | 5%Ag-MCSNs | -2.81667^*^ | 0.184 | 0.000 | -3.391 | -2.242 |
|  | 10%Ag-MCSNs | -12.40000^*^ | 0.184 | 0.000 | -12.974 | -11.826 |
| 1%MCSNs | 5%MCSNs | -1.18333^*^ | 0.184 | 0.000 | -1.758 | -0.609 |
|  | 10%MCSNs | -3.81667^*^ | 0.184 | 0.000 | -4.391 | -3.242 |
|  | 1%Ag-MCSNs | -.85000^*^ | 0.184 | 0.001 | -1.424 | -0.276 |
|  | 5%Ag-MCSNs | -2.43333^*^ | 0.184 | 0.000 | -3.008 | -1.859 |
|  | 10%Ag-MCSNs | -12.01667^*^ | 0.184 | 0.000 | -12.591 | -11.442 |
| 5%MCSNs | 10%MCSNs | -2.63333^*^ | 0.184 | 0.000 | -3.208 | -2.059 |
|  | 1%Ag-MCSNs | 0.333 | 0.184 | 0.548 | -0.241 | 0.908 |
|  | 5%Ag-MCSNs | -1.25000^*^ | 0.184 | 0.000 | -1.824 | -0.676 |
|  | 10%Ag-MCSNs | -10.83333^*^ | 0.184 | 0.000 | -11.408 | -10.259 |
| 10%MCSNs | 1%Ag-MCSNs | 2.96667^*^ | 0.184 | 0.000 | 2.392 | 3.541 |
|  | 5%Ag-MCSNs | 1.38333^*^ | 0.184 | 0.000 | 0.809 | 1.958 |
|  | 10%Ag-MCSNs | -8.20000^*^ | 0.184 | 0.000 | -8.774 | -7.626 |
| 1%Ag-MCSNs | 5%Ag-MCSNs | -1.58333^*^ | 0.184 | 0.000 | -2.158 | -1.009 |
|  | 10%Ag-MCSNs | -11.16667^*^ | 0.184 | 0.000 | -11.741 | -10.592 |
| 5%Ag-MCSNs | 10%Ag-MCSNs | -9.58333^*^ | 0.184 | 0.000 | -10.158 | -9.009 |

1. **Cytotoxicity**

| Time | Group | Mean (%) | Std. Deviation | Minimum | Maximum |
| --- | --- | --- | --- | --- | --- |
| 24 hrs | -ve control | 95.57 | 1.51 | 94.10 | 97.11 |
|  | +ve control | 94.71 | 0.74 | 93.87 | 95.25 |
|  | 1%MCSNs | 95.64 | 0.55 | 95.02 | 96.06 |
|  | 5%MCSNs | 95.99 | 0.24 | 95.72 | 96.18 |
|  | 10%MCSNs | 92.44 | 5.39 | 86.23 | 95.95 |
|  | 1%Ag-MCSNs | 93.71 | 3.87 | 89.24 | 96.06 |
|  | 5%Ag-MCSNs | 93.40 | 2.09 | 91.67 | 95.72 |
|  | 10%Ag-MCSNs | 92.28 | 1.67 | 90.39 | 93.52 |
| 72 hrs | -ve control | 93.17 | 1.01 | 92.01 | 93.87 |
|  | +ve control | 86.34 | 1.56 | 85.07 | 88.08 |
|  | 1%MCSNs | 91.82 | 4.05 | 87.15 | 94.33 |
|  | 5%MCSNs | 89.89 | 3.89 | 87.04 | 94.33 |
|  | 10%MCSNs | 84.15 | 0.61 | 83.45 | 84.61 |
|  | 1%Ag-MCSNs | 83.33 | 1.32 | 82.41 | 84.84 |
|  | 5%Ag-MCSNs | 87.08 | 2.71 | 85.07 | 90.16 |
|  | 10%Ag-MCSNs | 74.46 | 3.30 | 71.18 | 77.78 |
| 168 hrs | -ve control | 85.34 | 0.58 | 84.72 | 85.88 |
|  | +ve control | 80.01 | 3.13 | 77.66 | 83.56 |
|  | 1%MCSNs | 85.15 | 1.36 | 84.14 | 86.69 |
|  | 5%MCSNs | 75.73 | 2.83 | 73.26 | 78.82 |
|  | 10%MCSNs | 75.39 | 1.46 | 73.84 | 76.74 |
|  | 1%Ag-MCSNs | 78.97 | 3.39 | 75.35 | 82.06 |
|  | 5%Ag-MCSNs | 75.96 | 2.67 | 73.15 | 78.47 |
|  | 10%Ag-MCSNs | 72.03 | 1.14 | 70.72 | 72.80 |

| ANOVA | | | | | | |
| --- | --- | --- | --- | --- | --- | --- |
|  | | Sum of Squares | df | Mean Square | F | Sig. |
| 24 hrs | Between Groups | 45.093 | 7 | 6.442 | .948 | .499 |
|  | Within Groups | 108.772 | 16 | 6.798 |  |  |
|  | Total | 153.865 | 23 |  |  |  |
| 72 hrs | Between Groups | 734.415 | 7 | 104.916 | 15.164 | .000 |
|  | Within Groups | 110.699 | 16 | 6.919 |  |  |
|  | Total | 845.114 | 23 |  |  |  |
| 168 hrs | Between Groups | 477.234 | 7 | 68.176 | 12.982 | .000 |
|  | Within Groups | 84.026 | 16 | 5.252 |  |  |
|  | Total | 561.259 | 23 |  |  |  |

*Table 2: Multiple comparison using Dunnett posthoc test between experimental groups and negative control*

| Dunnett t (<control)^a^ | | | | |
| --- | --- | --- | --- | --- |
| Time | Group (I) | Group (J) | Mean Difference (I-J) | p-value |
| 24 hrs. | +ve control | -ve control | -0.85333 | 0.748 |
|  | 1%MCSNs | -ve control | 0.07000 | 0.883 |
|  | 5%MCSNs | -ve control | 0.42000 | 0.917 |
|  | 10%MCSNs | -ve control | -3.12667 | 0.285 |
|  | 1%Ag-MCSNs | -ve control | -1.85667 | 0.543 |
|  | 5%Ag-MCSNs | -ve control | -2.16333 | 0.476 |
|  | 10%Ag-MCSNs | -ve control | -3.28333 | 0.259 |
| 72 hrs. | +ve control | -ve control | -6.82667^*^ | 0.015 |
|  | 1%MCSNs | -ve control | -1.35000 | 0.653 |
|  | 5%MCSNs | -ve control | -3.27667 | 0.265 |
|  | 10%MCSNs | -ve control | -9.02333^*^ | 0.002 |
|  | 1%Ag-MCSNs | -ve control | -9.83667^*^ | 0.001 |
|  | 5%Ag-MCSNs | -ve control | -6.09333^*^ | 0.030 |
|  | 10%Ag-MCSNs | -ve control | -18.71000^*^ | 0.000 |
| 168 hrs. | +ve control | -ve control | -5.32667^*^ | 0.029 |
|  | 1%MCSNs | -ve control | -0.19333 | 0.848 |
|  | 5%MCSNs | -ve control | -9.60667^*^ | 0.000 |
|  | 10%MCSNs | -ve control | -9.95333^*^ | 0.000 |
|  | 1%Ag-MCSNs | -ve control | -6.36667^*^ | 0.010 |
|  | 5%Ag-MCSNs | -ve control | -9.37667^*^ | 0.000 |
|  | 10%Ag-MCSNs | -ve control | -13.31000^*^ | 0.000 |

**3-Radiopacity**

| **Descriptives** | | | | | | | | |
| --- | --- | --- | --- | --- | --- | --- | --- | --- |
| Radioopacity | | | | | | | | |
|  | N | Mean | Std. Deviation | Std. Error | 95% Confidence Interval for Mean | | Minimum | Maximum |
|  |  |  |  |  | Lower Bound | Upper Bound |  |  |
| 1% MCSNs | 10 | 9.9650 | .67360 | .21301 | 9.4831 | 10.4469 | 8.09 | 10.42 |
| 5% MCSNs | 10 | 9.8820 | .78864 | .24939 | 9.3178 | 10.4462 | 8.09 | 11.40 |
| 10%MCSNs | 10 | 9.9710 | 1.18163 | .37366 | 9.1257 | 10.8163 | 7.88 | 12.80 |
| 1%Ag-MCSNs | 10 | 10.3110 | .12467 | .03943 | 10.2218 | 10.4002 | 10.16 | 10.50 |
| 5%Ag-MCSNs | 10 | 10.3030 | .08908 | .02817 | 10.2393 | 10.3667 | 10.13 | 10.44 |
| 10%Ag-MCSNs | 10 | 10.4040 | .10885 | .03442 | 10.3261 | 10.4819 | 10.21 | 10.56 |
| Control | 10 | 10.2505 | .19652 | .06214 | 10.1099 | 10.3911 | 9.77 | 10.45 |
| Total | 70 | 10.1552 | .60829 | .07271 | 10.0102 | 10.3003 | 7.88 | 12.80 |

| **ANOVA** | | | | | |
| --- | --- | --- | --- | --- | --- |
| Radioopacity | | | | | |
|  | Sum of Squares | df | Mean Square | F | Sig. |
| Between Groups | 2.618 | 6 | .436 | 1.200 | .318 |
| Within Groups | 22.913 | 63 | .364 |  |  |
| Total | 25.531 | 69 |  |  |  |
