# Supplementary material for: Enhancing gutta-percha with silver mesoporous calcium silicate nanoparticles for advanced endodontic applications
Source: PLoS One. 2025 Aug 12;20(8):e0329435. doi: 10.1371/journal.pone.0329435 (PMC12342242; doi:10.1371/journal.pone.0329435)
Supplement: S1 Protocol — (DOCX) [file pone.0329435.s003.docx]

**S3 protocol of MTT assay procedure**

The used MTT assay Kit (OZ Biosciences MTT Cell Proliferation Assay Kit, USA) is composed of the following:

1. MTT solution 3-(4,5-dimethylthiazol-2yl) 2,5diphenyl tetrazolium bromide (MW=414) 1mL x 10vials.
2. Solubilization solution 50 mL x 2 bottles.

According to the following protocol, MTT assay was used to evaluate the cells' viability in response to MCSN and Ag-MCSN nanoparticles (for the three immersion times at 24h, 72h, and 168h) according to the International Standard ISO 10993-part 5 in 2009.

The cells (1 x 10^4^ cells) were cultured in a 96-well plate to a final volume of 100 μL complete culture medium per well. The plates were covered with a sterile parafilm, gently stirred, and incubated for 24 hours at 37 ^°^C, 5% CO_2_ atmosphere.

MTT3-(4,5- dimethylthiazol - 2-yl) - 2,5- diphenyltetrazolium bromide was dissolved in Dulbecco’s phosphate buffered saline (DPBS) at pH 7.4 (5 mg/mL) to create MTT solution. Through a 0.2-m filter and into a sterilized, light-protected container, this solution was filtered and sterilized. MTT solution was kept in a dark, − 20^°^ C environment until analysis. In a ventilated fume hood, a solubilization solution was made using 40% (v/v) dimethylformamide (DMF) and 2% (v/v) glacial acetic acid (Sigma Aldrich Co. USA). This solution’s pH was raised to 4.7 and Sodium dodecyl sulfate (SDS) (Sigma Aldrich Co. USA). (16% [w/v]) was added. Storage of the solubilization solution at room temperature to stop SDS from precipitating. Cell suspensions containing MCSNs and Ag-MCSNs nanoparticles were seeded onto 96-well plates (100 μl/well) (BRAND, 40072, Germany) and cultured at 37 ◦C in a humid incubator with 5% CO_2_ for 24 h, 72 h, and 168 h. Cells cultured without nanoparticles were used as a negative control. A 10 μl MTT solution was added to each well, and it was then incubated at 37 ◦C for 1–4 h with a final concentration of 0.45 mg/mL. Following incubation, 100 μl of solubilization solution was added to the formazan crystals, and an optical multi plate reader was used to detect the absorbance at 570 nm (Spectramax 190, Molecular Devices, USA). As controls, cells that weren’t exposed to the nanoparticles were employed. Three copies of the optical density (OD) findings were collected. The percentage of cell viability was calculated using the following equation:

% Viability = Mean OD sample/Mean OD blank × 100 (1)
