# Supplementary material for: Enhancing gutta-percha with silver mesoporous calcium silicate nanoparticles for advanced endodontic applications
Source: PLoS One. 2025 Aug 12;20(8):e0329435. doi: 10.1371/journal.pone.0329435 (PMC12342242; doi:10.1371/journal.pone.0329435)
Supplement: S1 Fig — (DOCX) [file pone.0329435.s004.docx]

**S4 Fig. of SEM for G.P.**

**
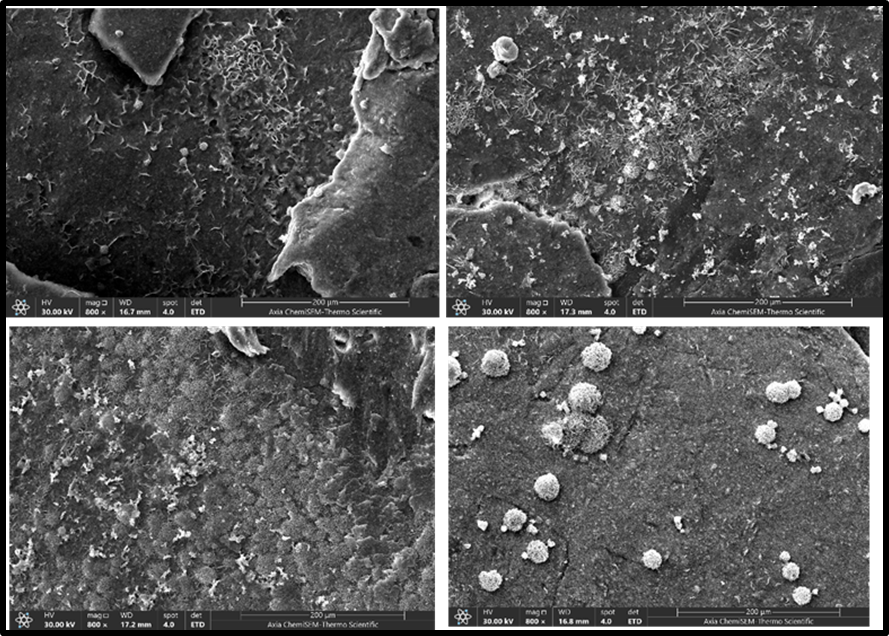
**

Figure : Scanning electron microscope (SEM) for Gutta-percha after soaking in SBF.
