# Supplementary material for: Enhancing gutta-percha with silver mesoporous calcium silicate nanoparticles for advanced endodontic applications
Source: PLoS One. 2025 Aug 12;20(8):e0329435. doi: 10.1371/journal.pone.0329435 (PMC12342242; doi:10.1371/journal.pone.0329435)
Supplement: S2 Fig — (DOCX) [file pone.0329435.s005.docx]

**S5 Fig. of XRD spectra for G.P. mixed with MCSNs and Ag-MCSNs**

**
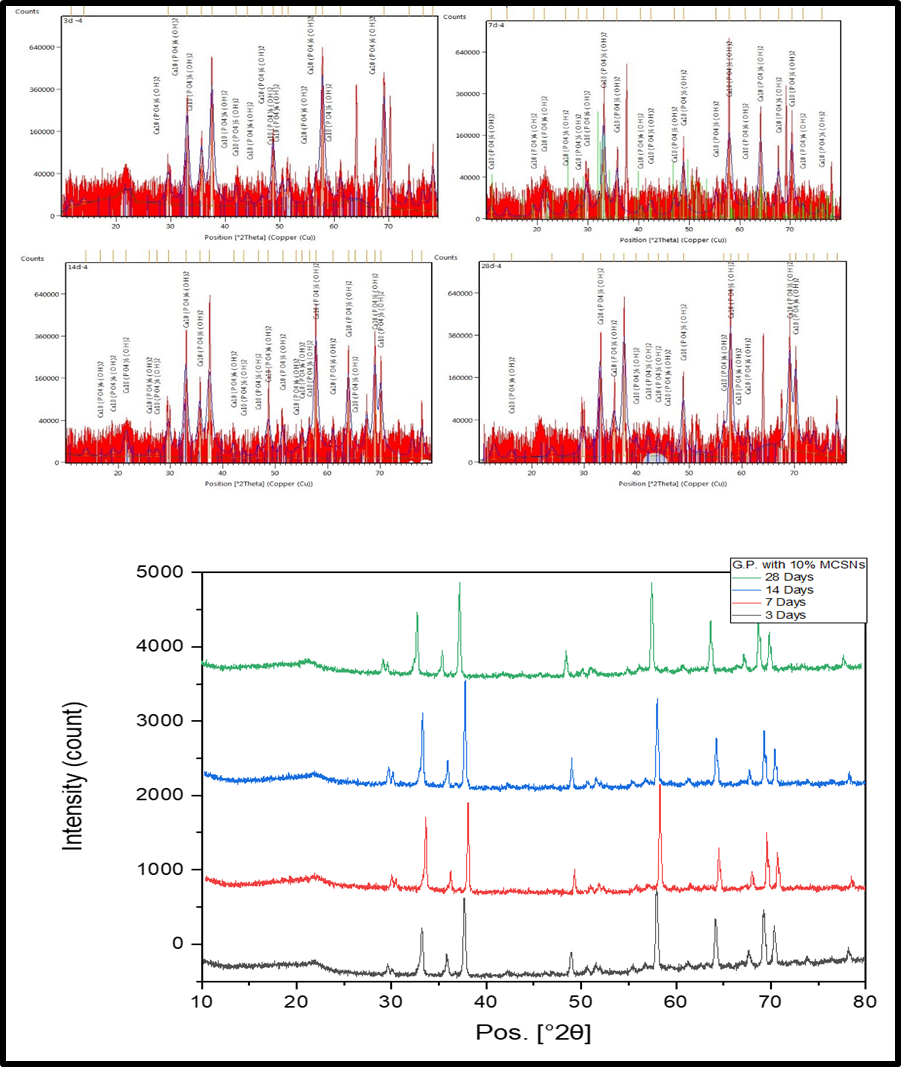
**

Figure: X-ray diffraction (XRD) for Gutta-percha after mixing with 10% MCSNs filler. (A) after 3days , (B) after 7 days , (C) after 14 days , (D) after 28 days using X'pert highscore software – 23 & (E) XRD different days.


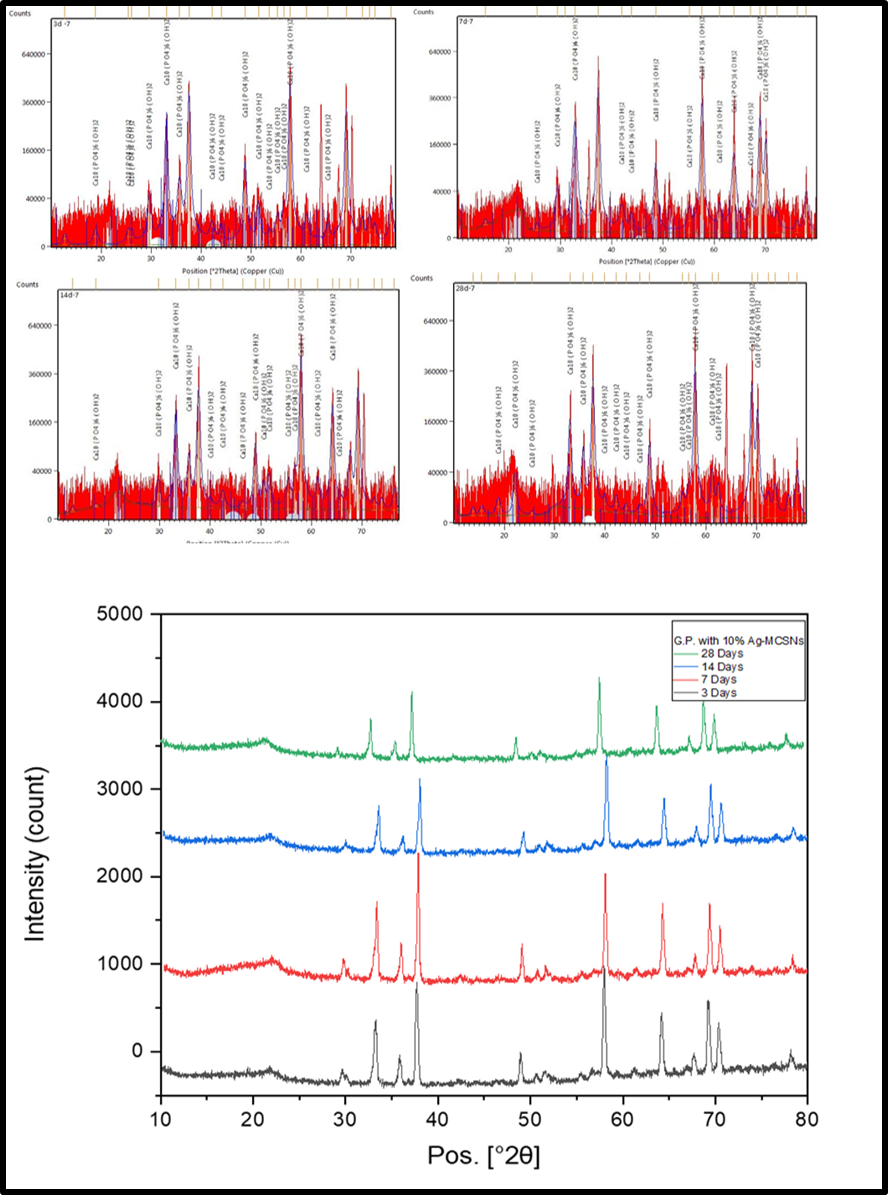


Figure :X-ray diffraction (XRD) for Gutta-percha after mixing with 10% Ag-MCSNs filler. (A) after 3days , (B) after 7 days , (C) after 14 days ,(D) after 28 days using X'pert highscore software – 23 & (E) XRD different days.
